# Investigating Consumer Preferences for Production Process Labeling Using Visual Attention Data

**DOI:** 10.3390/bs9070071

**Published:** 2019-07-01

**Authors:** Xuan Wei, Hayk Khachatryan, Alicia L. Rihn

**Affiliations:** 1 Mid-Florida Research and Education Center, University of Florida, Apopka, FL 32703, USA; 2 Food and Resource Economics Department and Mid-Florida Research and Education Center, University of Florida, Gainesville, FL 32611, USA

**Keywords:** visual fixations, total visit duration, visual fixation duration, neonicotinoid labels, second-price auctions, gaze cascade effect

## Abstract

A second-price auction with eye movement recordings was used to investigate consumer preferences for labels disclosing the presence and absence of specific types of insecticides and to explore the relationship between visual attention and consumer purchasing behaviors. Findings contribute to the literature in the following ways. First, visual attention pattern was endogenously determined by personal knowledge and pollinator conservation activities. Less knowledgeable or less engaged participants fixated more and for longer durations on the product as a whole rather than other information. Secondly, the first and last gaze cascade effect was confirmed by identifying a significant negative impact of participants’ first and last gaze visits on neonicotinoid labels on their bid values. Third, new evidence was added to the existing literature that the link between visual attention and consumer valuation and preference may be weak. Our results suggest that visual attention could provide useful information toward understanding participants’ bidding behaviors; however, evidence indicates that visual attention measures may not be directly linked with decision making.

## 1. Introduction

Visual attention, measured by eye tracking, increasingly attracts behavioral and experimental economists who investigate consumer preferences and willingness to pay (WTP) for products with different attributes. When modeling consumer preferences for product attributes in choice experiments and experimental auctions, the conventional economic approach assumes that consumers process all of the presented information and tradeoff among different product alternatives [1]. However, recent evidence from marketing literature and consumer neuroscience literature (a growing research area) suggests that consumers apply heuristic decision rules in processing product information. For instance, consumers might not attend to all product attributes, i.e., attribute non-attendance [2,3,4,5,6,7,8,9,10,11,12,13,14,15,16,17,18,19]. Some studies show consumers are more likely to select the choice that attracted their attention/gaze first or last, i.e., gaze cascade effect [20,21,22,23]. Other studies demonstrate consumers primarily evaluate products with specific attributes that are important to them (i.e., attribute focus or lexicographic choice). These findings implicitly acknowledge that visual attention is a crucial measure that should be taken into account when analyzing consumer preferences [24]. 

There is a new trend of using eye-tracking technology to obtain information on attribute non-attendance in choice experiments. Given that the primary approach when exploring consumer choice and preference heterogeneity is through modeling product attributes, incorporating visual attention into attribute non-attendance (called visual attribute non-attendance by Van Loo et al. (2018) [18]) has attracted considerable research interest among experimental economists. Meanwhile, other important visual attention patterns that are well documented in marketing literature (such as the gaze cascade effect, centrality bias, and attribute focus) have received relatively little attention in economic fields.

To address this research gap, in this manuscript eye-tracking was combined with non-hypothetical second-price auctions to elicit participants’ preferences and WTP for gardening products in central Florida. In this study, the attribute focus (Hess et al. (2010) [25] termed it lexicographic choice) and first and last gaze cascade effects were empirically tested while consumer preferences for labels disclosing the presence and absence of specific types of insecticides (neonicotinoids) were examined. The endogeneity issue of visual attention due to unobserved individual characteristics (such as an existing interest in pollinator conservation or concerns about potential environmental impacts of neonicotinoid use) was considered. Lastly, how visual attention patterns may help explain consumer WTP (in the form of bid values) for different neonicotinoid labeling formats and information framing were addressed. 

The rest of the paper is organized as follows. In Section 2, a brief overview of relevant eye tracking studies is provided followed by the hypotheses and study contributions. Section 3 introduces research methodology. It consists of a detailed description of the experimental design including an areas of interest (AOI) explanation, visual attention measures, and data description as well as the theoretical and empirical framework. Results are discussed in Section 4. The last section concludes by discussing the implications and limitations of the study. 

## 2. Review of Related Literature and Hypothesis

The literature discussed in this section begins with an overview of eye tracking studies, followed by endogeneity issues, knowledge, and participant involvement. Lastly, visual attribute attendance and the gaze cascade effect will be discussed. Throughout the literature review, the relevant hypotheses will be presented.

Eye tracking as an experimental tool has recently gained traction in several disciplines. Eye tracking has been extensively used in marketing literature to address visual marketing strategies related to brands and product attributes [26,27]. Numerous studies have shown that visual attention patterns (e.g., fixations and duration) can be predictive of choice since a longer duration or more fixations can lead to higher choice likelihood [20,28,29,30,31]. Meanwhile, visual attention patterns can be affected by individual values, which reflect personal preferences. Hence, the degree to which visual attention plays a role in determining how individuals make choice decisions and reveal preferences is of great interest to economists. 

Visual attention measurements (e.g., fixation counts, fixation durations) have recently been utilized as explanatory variables in econometric models to explore the correlations between visual attention and individual choice behavior [19,29,32,33,34]. However, the visual attention estimate effects on individual choice are likely biased due to potential endogeneity issues related to the visual attention variable(s) [35]. For instance, if an unobserved personal characteristic affects both the individual’s visual attention and plant purchasing decisions (the dependent variable in this research), the estimated effect of the visual attention variable could be contaminated by the effect of the unobserved characteristic(s) resulting in bias. 

Currently, very few studies utilize eye tracking and account for endogeneity. Takahashi et al. (2018) [35] was an exception who combined eye tracking with a randomized controlled trial (RCT) to explicitly address this issue. Respondents’ (unobserved) environmental concerns and exposure to certification information influenced both their visual attention and purchasing behaviors. Furthermore, a sustainable graphic representation attracted more visual attention and increased purchase likelihood by 22%. This suggests that endogeneity should be acknowledged and addressed (when possible) in studies utilizing eye tracking.

Participants’ existing knowledge has been shown to influence behavior. Frequently, knowledge is measured either as subjective or objective knowledge [36]. Subjective knowledge reflects what the consumer thinks s/he knows while objective knowledge is their actual knowledge [37,38]. Subjective knowledge is quantified using a scale where the respondent indicates his/her self-perceived knowledge while objective knowledge is frequently revealed using quiz questions [37,39]. In general, consumers who report high subjective knowledge frequently exhibit low objective knowledge but are quick to project their self-perceived knowledge on the topic [39,40]. Previous research suggests that both types of knowledge potentially aid in lessening the endogeneity issue and should be considered in studies [37]. 

Beyond knowledge, participant involvement with topics related to the study’s focus likely also impact their behavior. Involvement with products has been shown to impact consumer perceptions, product purchasing behavior, motivations, comprehension, and visual attention to relevant information [41,42,43,44]. Specifically related to eye tracking, several studies have found that consumers’ visual attention increases for stimuli that is directly related to their interests [43,45]. One measure of involvement in pollinator health is conservation activities. Previous studies have addressed WTP for pollinator conservation at a national level rather than viewing current household conservation-related activities. For instance, studies estimate that UK and US consumers are willing to donate money or pay taxes to support pollinator conservation [46,47,48]. Yet, studies highlight the importance of household pollinator conservation activities to increase habitat availability and foraging opportunities [49,50]. The participation in conservation activities at the household level may indicate increased interest and concern with overall pollinator health. This implies that including household pollinator conservation activities could impact behavior but has yet to be included in empirical analysis. 

Given that endogeneity can bias results, the inclusion of additional explanatory variables is one means of addressing this issue (i.e., endogeneity caused by missing variables). Knowledge and involvement (measured by participation in pollinator conservation activities) largely reflect individuals’ increased interest in pollinator health and concerns about neonicotinoids. To date, the two factors have not been combined in empirical analysis but may provide valuable insights into consumer behavior. One could argue that the two factors are related in that someone who is more knowledgeable is likely to be more involved than someone who is less knowledgeable. Hence, the following hypotheses were tested:

**Hypothesis 1.**  *Visual attention is endogenously determined by individual knowledge and activities related to neonicotinoid insecticides and pollinator conservation that reflect individual interests or concerns about pollinator conservation.*

**Hypothesis 2a.**  *A higher level of knowledge about the potential impact of neonicotinoids will lead to greater visual attention to the associated attribute (e.g., labels disclosing the absence or presence of neonicotinoids).*

**Hypothesis 2b.**  *A higher level of involvement with pollinator conservation activities will lead to greater visual attention to the associated attribute (e.g., labels disclosing the absence or presence of neonicotinoids).*

More recently, eye tracking has been used to obtain information on attribute attendance/non-attendance in choice experiments [17,18,45,51,52,53,54]. These studies attempt to establish a direct link between visual attention and attribute attendance/non-attendance and further infer the relationship between visual attention and individual preference. For instance, Krucien et al. (2017) [53] showed that the relationship between visual attention and individuals’ preferences depends on the product attribute type. Preferences for “harder to process” attributes were more influenced by changes in visual attention relative to “easier to process” attributes. Behe et al. (2014) [45] determined that visually attention increases on information that is important to consumers. On the other hand, Balcombe et al. (2017) [52] and Van Loo et al. (2018) [18] reported weaker relationships between visual attention and individual preferences since accounting for visual attribute non-attendance did not significantly influence WTP. Balcombe et al. (2017) [52] revealed that visual attention data was not a good indicator of individual stated attendance. Eye tracking data was useful in understanding attribute non-attendance but less informative with regard to WTP. Similarly, Van Loo et al. (2018) [18] concluded that not all visually ignored attributes were truly ignored based on the observed respondents’ choice behaviors and using visual attention to identify attribute attendance was not trivial.

While it is relatively new in the field of economics, the use of eye tracking has been widely applied in the fields of marketing and psychology. Studies have consistently shown subjects pay more attention to chosen alternatives than non-chosen alternatives [10,22,55,56]. Hess and Hensher (2010) [10] found that consumers attached less importance to certain attributes and persistently selected the alternatives that had better specific attributes. Meiϐner et al. (2016) [57] showed that high-valued alternatives or important attributes received increased attention. Hess et al. (2010) [10] termed this pattern as lexicographic choice, and Meiϐner et al. (2016) [57] framed it as an alternative focus/attribute focus and investigated how attribute and alternative focus potentially affected subjects’ final choice. 

In parallel, information literature has recognized the positive-negative asymmetry in human behaviors when evaluating the impact of information on consumer valuations of a good [58,59]. Numerous studies confirmed that negative information has a stronger impact on consumer behavior than positive information [60,61,62,63]. Building upon the asymmetry of the information effect (i.e., negativity effect), the present study investigates the link between attribute focus and individual bidding behavior by testing the symmetry of attribute focus. Specifically,

**Hypothesis 3a:**  *Greater attention on attributes communicating positive environmental information (e.g., label disclosing the absence of neonicotinoids) will increase consumer’s WTP.*

**Hypothesis 3b:**  *Greater attention on attributes communicating negative environmental information (e.g., label disclosing the absence of neonicotinoids) will decrease consumer’s WTP.*

In addition, Shimojo et al. (2003) [22] proposed the concept of gaze cascade effect described as an increased likelihood that observers’ gazes were directed toward the chosen object. In subsequent studies, Changizi and Shimojo (2008) [21], Simion and Shimojo (2006) [23], and Atalay et al. (2012) [20] demonstrated a link between observers’ final gaze and the chosen object. Meanwhile, Reutskaja et al. (2011) [64], Fisher and Rangel (2014) [65], and Meiϐner et al. (2016) [57], showed that the feature on which participants first fixated was more likely to be chosen. These results suggest that the first and last gaze might influence choice. In this study, gaze cascade effects were tested by investigating whether the first and last gaze fixation on a label increased or decreased participants’ WTP for that attribute.

**Hypothesis 4a:**  *Consumer’s first gaze fixation on a particular attribute (i.e., label) will increase (decrease) the consumer’s bid value and WTP if the attribute communicates positive (negative) environmental information.*

**Hypothesis 4b:**  *Consumer’s last gaze fixation on a particular attribute (i.e., label) will increase (decrease) the consumer’s bid value and WTP if the attribute communicates positive (negative) environmental information.*

By testing the above-mentioned hypotheses, this manuscript contributes to the literature in several ways. First, hypotheses 1 and 2 verify if consumers’ visual attention patterns are endogenously determined by testing the influence of visual attention, knowledge, and pollinator conservation activities on bid values. Secondly, the manuscript addresses if increased visual attention to important attributes (such as neonicotinoid labels) carries through to the final decision (hypotheses 3a and 3b). According to the noncompensatory processing model in consumer product choice literature, consumers either screen out alternatives that have an undesired feature/attribute or consider only those that have a desired feature/attribute [57,66]. By evaluating visual attention to more/less desirable features, one can assess how this information influences bidding. Visual attention provides useful information regarding participants’ decision-making behavior; however, it is not sufficient to identify whether the information was ignored or entered into the decision-making process [18,67]. In other words, the relationship between visual attention and consumer preferences may be weak [52]. Here, the link is investigated. Lastly, using a left-right paradigm (in contrast to horizontal paradigm), evidence addressing the gaze cascade effect on participants’ valuation is explored. Currently, Atalay et al. (2012) [20], Gilbride and Allenby (2004) [66], and Meiϐner et al. (2016) [57] all found initial gaze had minimal effects on product choice when items were placed in a horizontal paradigm. 

## 3. Research Methodology

To address the research goals and investigate the hypotheses, an eye-tracking experiment was combined with non-hypothetical second price auctions to elicit participants’ preferences and WTP for gardening products in central Florida. 

### 3.1. Experiment Design

Based on the 2014 NASA Survey results on sales value, six of the most popular pollinator friendly plants—Impatiens, Marigold, and Pentas (annual bedding plants) and Dianthus, Chrysanthemum, and Salvia (three perennial plants) were selected for bidding items (Table 1). According to 2014 NASS Survey results, the sales values of Impatiens, Marigold and Pentas were ranked the 5th, 7th and 25th of all annual bedding plants while the sales values of Chrysanthemum, Salvia and Dianthus were ranked the 1st, 5th, and 6th of all perennial plants. The same plant images were used throughout the experiment to control for aesthetic variances. The annual bedding plants were in 4-inch containers, while perennial plants were in 1-gallon containers. Importantly, three different types of annual bedding plants and three different perennial plants were used to partially control for other attributes that could be important to consumer’s purchase decision (such as flower type, color, and size). 

**Figure 1:**
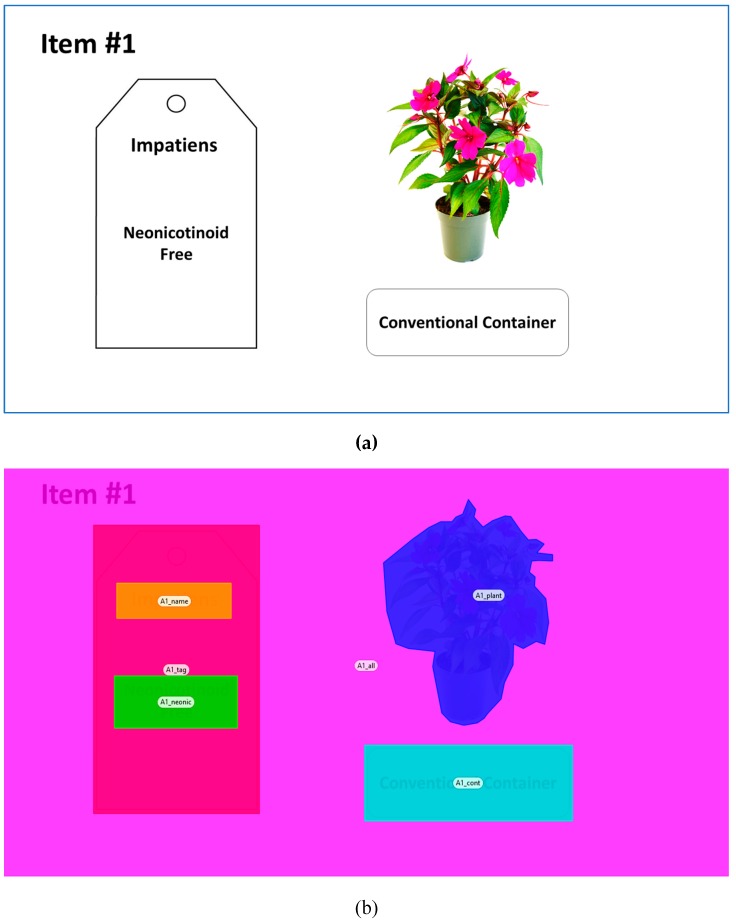
(**a**) An example of a product scenario presented during the experimental auctions. (**b**) An example of areas of interests used to extract visual attention measures after the auction experiments. Note: The areas of interest named A1_name, A1_neonic, A1_plant, and A1_cont indicate the plant name, labeling, plant image, and the container type, respectively.

Several other attributes were included in the product design (Table 1). Two distinct neonicotinoid labeling categories (absent/present) were used along with different forms (text vs. logo) and different text framings (Treated with Neonicotinoids vs. Protected from Problematic Pests by Neonicotinoids). Neonicotinoid-Free (text) and Bee Better Certified^TM^ (logo) communicated the absence of neonicotinoids (Detailed information about Bee Better Certified ^TM^ Production Standards is available at https://beebettercertified.org/docs). Treated with Neonicotinoids and Protected from Problematic Pests by Neonicotinoids phrases indicated the presence of neonicotinoids during production and were adopted from Home Depot plant tags. Lastly, biodegradable containers were included as an alternative to conventional containers given a recent rise of biodegradable or compostable container options [68] and consumer demand for “green” or “sustainable” products [68,69]. Figure 1a displays an example of one of the auctioned items.

Four areas of interests (AOIs) were defined to collect visual attention data on the previously discussed attributes. The AOIs included the plant name, the neonicotinoid labels, the plant image, and the container type (Figure 1b). The overall display followed standard binary-choice designs in existing consumer neuroscience literature [70,71,72]. The left–right design circumvented the central fixation bias that occurs because of the natural initial visual response regardless of how the informative is displayed [20,73,74]. 

In practice, many eye-tracking studies look for patterns based on fixations and saccades. Balcombe et al. (2017) [52] regarded fixations as a good indicator of visual attention partially because little visual information can be obtained during saccades [75]. In this study, five different fixation measures were used to capture different aspects of participants’ visual attention including: fixation count (FC), total fixation duration (TFD), time to first fixation (TFF), first fixation, and last fixation. The first three measures (FC, TFD, TFF) are extensively used in eye tracking studies analyzing the effect of visual attention on choice outcomes [19,33,34,45,76]. Following Atalay et al. (2012) [20], first fixation (i.e., initial gaze) and last fixation (i.e., final gaze) were also captured to test the gaze cascade effect on an individual’s preference. However, in contrast to Atalay et al. (2012) [20], who measured the duration of the first and last fixation, in this study the two metrics were measured based on whether it was the first or last fixation on an AOI. Specifically, the first and last fixations are binary variables equal to one if a participant fixated first or last on a specific AOI and zero otherwise. 

### 3.2. Data Collection and Sample Description 

The experiment was conducted in an experimental laboratory in central Florida in November 2017. In total, 15 auction sessions with 75 participants were conducted with 53 participants having their eye movements recorded. Given the focus on visual attention in this manuscript, only the 53 participants with visual attention data were used in the analysis. It is worth noting that in eye tracking studies, sample sizes are usually smaller than traditional consumer behavior studies given the in-person participation requirements and budget constraints. For example, previous eye tracking studies utilized samples between 40 and 81 participants [19,51,64,76,77]. In this study, each participant received 25 dollars as compensation.

Participants signed a consent form (approved by the Institutional Review Board (IRB approval: IRB201601783)) and received brief instructions including an overview of the second price auction procedures. Additionally, the eye tracking cameras were calibrated to the participants at the eye tracking computers. A stationary Tobii X1 Light Eye Tracking camera on the base of a 22-inch (1920 × 1080 pixel resolution) computer monitor captured participants’ visual attention during the experiment (Tobii Studio Software 3.4.8. (Tobii AB (publ), Danderyd, Sweden)). There were two auction rounds per auction session where participants bid on 14 annual bedding and 14 perennial plants. Participants carefully viewed each plant, wrote their bids on a bidding sheet, and then move to the next item. During the course of the auction, they could not move backwards or revisit an item. In each auction session, one annual and one perennial plant were randomly selected as the winning items after all bids were submitted. The winning participants of the two products were announced and were informed of the market price (i.e., second highest price) which was subtracted from the compensation at the completion of the experiment. Participants were instructed that they could only win one item to control for diminishing marginal utility. A participant would be randomly assigned to one product by flipping a coin if s/he won both products.

Participants’ sociodemographic characteristics are listed in Table 2. About 15% of the participants were male and the average age was about 53 years old. This was partially due to female consumers and older age groups being the core consumers of plants [78]. Participants’ average household income was between $40,000–$59,000. On average, they visited retail centers six times per year to purchase plants and spent less than 40 dollars on plants each visit. 

To assess individual knowledge heterogeneity, questions elicited information on individual knowledge, perceptions, and activities related to pollinator conservation. Participants indicated if they had heard about neonicotinoid pesticides (Yes/No), followed by how knowledgeable they were about neonicotinoid pesticides, four quiz questions to reveal actual knowledge, and their involvement in pollinator conservation activities. 

Overall, 23% of the participants had heard about neonicotinoids while 11.3% self-perceived themselves as knowledgeable (Table 2). This is consistent with the national level of public knowledge about neonicotinoids indicating reliability [79,80]. Quiz questions quantified participants’ ability to correctly identify pollinator attractive plants. Only 6% of the participants correctly answered all of the questions, 33% correctly answered three of the four questions, and 8% incorrectly answered all of the questions. For analysis, participants were defined as knowledgeable about neonicotinoid if and only if they had heard about neonicotinoid pesticides and selected 4 or higher on the knowledge scale. Additionally, a binary actual/real knowledge variable was generated where respondents who correctly answered three or more quiz questions were categorized as “knowledgeable about pollinator attractive plants” while those who correctly answered two or less were considered not knowledgeable. As discussed in the knowledge literature, the use of self-reporting scales and quiz questions are standard practice when measuring subjective and objective/actual knowledge of topics of interest [37,39]. 

Regarding participants’ actual involvement in pollinator conservation activities, they selected the actions they were currently taking to improve pollinator health from a pre-defined list. The list included: (a) plant selection to feed adults, (b) plant selection to feed larvae/young, (c) decrease or do not use pesticides, (d) add features to aid pollinator insects (brush piles, water sources), (e) source plants locally, (f) primarily buy native plants, (g) primarily buy plants that are labeled as helpful to pollinators, and (h) primarily buy flowering plants. A binary variable was created where engagement in more than one activity was categorized as “actively engaged” (i.e., involved) in pollinator conservation. 81% of the participants are actively engaged in pollinator conservation while 19% indicated that they did not participate in pollinator conservation activities. 

The pairwise correlation between the perceived knowledge about neonicotinoids and perceived knowledge about pollinator attractive plants, between perceived knowledge about pollinator attractive plants and conservation activity was about 0.433 and 0.437, respectively, indicating self-perceived knowledgeable participants tended to be more involved in pollinator conservation activities. However, there was a gap between participants’ perceived knowledge and revealed knowledge about pollinator attractive plants as the correlation between these two variables was only about 0.121 (Appendix A Table A1). In general, we did not see significant differences in participants’ perceived knowledge and involvement in pollinator conservation activities across gender and age groups (see Appendix A Table A2). However, participants who perceived themselves as knowledgeable about neonicotinoids tended to be older than the not knowledgeable group (paired-*t* test statistic is −2.89 with a *p*-value of 0.01). This may be attributable to knowledgeable participants’ longer experience with gardening and handling garden pests. 

### 3.3. Model 

Participants’ value for plants with different attributes was collected using the incentive compatible second-price auction [81]. Let $v_{i}$ be the value individual i placed on a plant. An individual submits a bid $b_{i}$, which is the highest amount that s/he is willing to pay for the item against N rival bidders (within the same session). Participants were informed that the market price p, which in this case is the second highest bid, is determined after all the bids were submitted. Therefore, the goal of an individual bidder is to submit a bid $b_{i}$ to maximize expected utility given by:

(1) $$E\left(U_{i}\right)=\overset{b_{i}}{\underset{\underline{p_{i}}}{\int }}U_{i}\left(v_{i}-p\right)g_{i}\left(p\right)dp+\overset{\bar{p_{i}}}{\underset{\underline{b_{i}}}{\int }}U_{i}\left(0\right)$$

where the market price $p\in \;\left[\underline{p_{i}}.\bar{p_{i}}\;\right]$ is a random variable with a probability density function of $g_{i}\left(p\right)$ as individual i does not know how much other bidders will bid and thus what p will be. The first integral is the utility when individual i bids higher than the market price and actually wins the auction. The second integral is the utility when individual i bids lower than the market price and loses the auction. Normalizing U(0)=0, the optimal bid is obtained by taking derivative with respective to $b_{i}$ and solving the following first order condition.

(2) $$\frac{\partial E\left(U_{i}\right)}{\partial b_{i}}=U_{i}\left(v_{i}-b_{i}\right)g_{i}\left(b_{i}\right)=0$$

The bidder’s expected utility is maximized when s/he submits a bid equal to his or her value/WTP for the plant. According to [82], this optimal value does not depend on individual’s risk preference, initial income level, the number of rival bidders, as well as other bidders’ bidding strategy. 

Based on the theoretical framework, one can assume that a latent variable $b_{ij}^{*}$ exists that represents the participants’ true WTP for a plant j offered in the auction session. In second-price auctions, it is common to observe some zero bids while other bids take strictly positive values. A random effects tobit model was used to analyze the data. Following [83], $b_{ij}^{*}$ is assumed to follow a linear unobserved effects model. The latent variable $b_{ij}^{*}$ is related to the observed bid $b_{ij}$ by

(3) $$b_{ij}=\{\begin{array}{cc} 0 & if\;b_{ij}^{*}\leq 0\\ b_{ij}^{*}=x_{ij}\beta +c_{i}+u_{ij} & if\;\;b_{ij}^{*}>0 \end{array}$$

$x_{ij}$ is a vector of plant attributes and individual characteristics that influence consumers’ bidding price. Particularly, in this model, different visual attention measures are incorporated to explore the correlations between visual attention and product valuation. First fixation (FF_neonic) and last fixation (LF_neonic) were interacted with neonicotinoid labels (neonicotinoid-free text label, neonicotinoid-free logo label and neonicotinoid-treated text label) to test if first and/or last fixated alternatives may impact participants’ choice and WTP for neonicotinoid related labels (i.e., cascade effect). Alternatively, the proportion of fixation counts on specific labels, aiming to reflect the relative importance of a specific attribute, were interacted with its corresponding plant attribute to test how attribute focus may affect participants’ bid values and their WTP. Built upon the concept of relative attribute importance from [57,84], the proportion of fixation counts on attribute *j* were computed as follows:

(4) $$R\_FC=\frac{FC\_j}{\sum_{j=1}^{4}FC\_j}$$

where j={1, 2, 3, 4} and belongs to one the four major AOIs: plant name, neonicotinoid label, plant image and container type. 

In addition to general sociodemographic characteristics such as age, gender, income and education, three variables related to individual knowledge and pollinator conservation activities were included in the regression model to approximate unobserved interests and concerns. The three binary variables include perceived knowledge about neonicotinoid, real knowledge about pollinator attractive plants, and pollinator conservation activities (defined in Section 3.2: Data Collection and Sample Description). The variables were further interacted with visitation attention on neonicotinoid labels (FF_neonic, LF_neonic, R_FC_neonic) to capture differentiated preferences across heterogeneous groups (TFD was used for robustness check. Specifically, FF_neonic and LF_neonic stood for first and last fixation on neonicotinoid labels while R_FC_neonic reflected the importance of fixation counts on neonicotinoid labels among all four AOIs. $c_{i}$ is the unobserved individual-specific random effects varying across each individual i but not plant j. The random error term $u_{ij}$ has a normal distribution with a zero mean and variance $\sigma_{u}^{2}$.

## 4. Results

### 4.1. Visual Attention Summary

Table 3 summarizes the visual attention metrics. Across the four AOIs, plant image received the most visual attention, followed by the neonicotinoid label. In general, the plant image captured the first fixation and more visual attention than other attributes. This was consistent with the effect of visual saliency on predicting attention captured [24,85,86,87]. On average, participants fixated twice and spent less than one second on the neonicotinoid labels. Plant name received the least visual attention. Given that the first fixation and last fixation were binary variables, the mean values of the first/last fixation reflect the proportion of the participants who fixated first/last on each AOI. More than 36% of the participants had their first fixation on the plant image, and about 18% first fixated on the neonicotinoid labels. On the other hand, more than 42% of participants’ last fixation was on the container type, and only 10% to neonicotinoid labels. Container type captured more last fixations which may reflect the physical location of the container type (bottom right). Participants may have naturally fixated in that area during the course of moving to the next plant image. 

**Figure 2:**
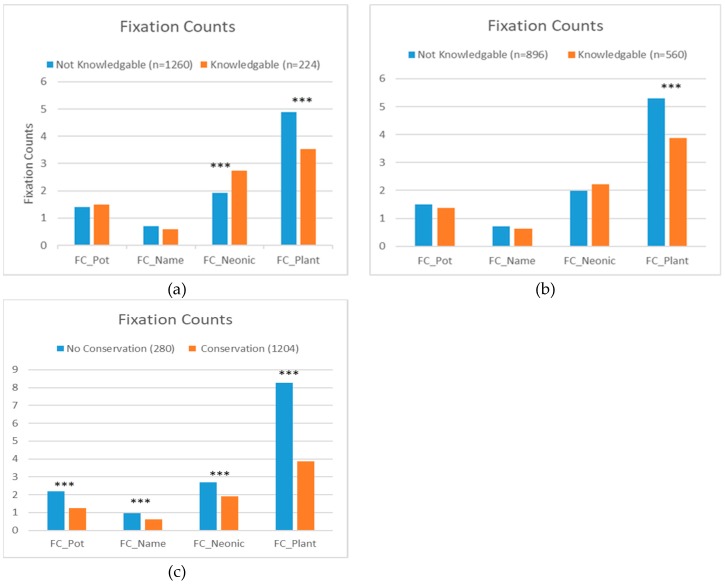
Fixation counts patterns by different groups. (**a**) by perceived knowledge about neonicotinoids; (**b**) by knowledge about pollinator attractive plants; (**c**) by conservation activity. Notes: *** indicates whether the mean difference between participants in different groups are statistically significant (*p* < 0.01) based on a pairwise *t*-test. In Figure 2a, respondents were defined as knowledgeable about neonicotinoids if they had heard about neonicotinoids and self-reported higher levels of knowledge about neonicotinoids (i.e., selected 4 or higher on the seven-point Likert knowledge scale). In Figure 2b, grouping of knowledgeable and not knowledgeable groups was based on participants’ answers to quiz questions. A respondent is defined as knowledgeable about pollinator attractive plants if he/she correctly answered to at least three out of the four quiz questions. In Figure 2c, grouping of pollinator conservation groups was based on participants’ self-reported actions. A respondent is defined as involved in conservation efforts if he/she is currently taking at least one action to improve pollinator health in his/her landscape, yard or garden. Such actions include (a) plant selection to feed adults, (b) plants selection to feed larvae/young, (c) decrease/do not use pesticides, (d) add features to aid pollinator insects (brush piles, water sources, etc.) (e) Source plants locally, (f) primarily buy native plants, (g) primarily buy plants that are labeled as helpful to pollinators, (h) primarily buy flowering plants.

**Figure A1:**
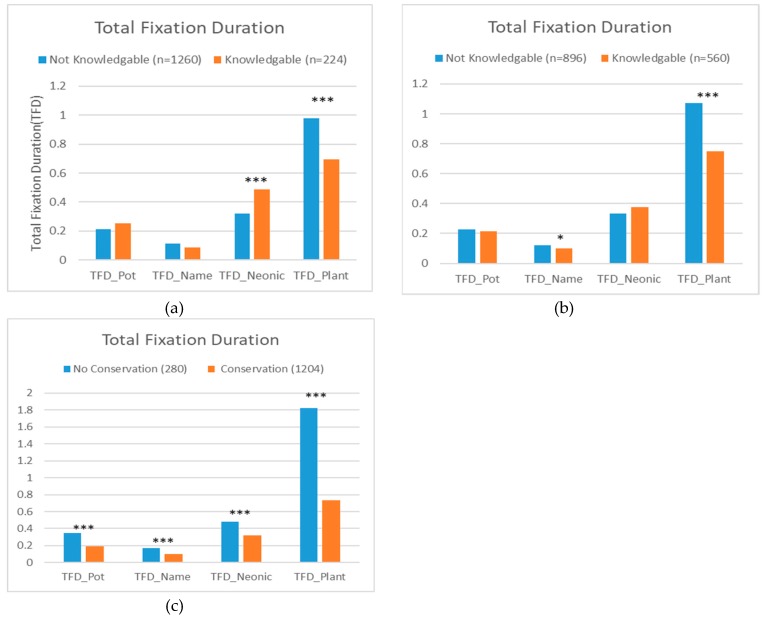
Total fixation duration patterns by different groups. (**a**) by perceived knowledge about neonicotinoids; (**b**) by knowledge about pollinator attractive plants; (**c**) by conservation activity. Notes: ***, ** and * indicates whether the mean difference between participants in different groups are statistically significant (* *p* < 0.10; ** *p* < 0.05; and *** *p* < 0.01) based on a pairwise *t*-test. In Figure A1-a, respondents were defined as knowledgeable about neonicotinoids if they had heard about neonicotinoids and self-reported higher levels of knowledge about neonicotinoids (i.e., selected 4 or higher on the seven-point Likert knowledge scale). In Figure A1b, grouping of knowledgeable and not knowledgeable groups was based on participants’ answers to quiz questions. A respondent is defined as knowledgeable about pollinator attractive plants if he/she correctly answered to at least three out of the four quiz questions. In Figure A1c, grouping of pollinator conservation groups was based on participants’ self-reported actions. A respondent is defined as involved in conservation efforts if he/she is currently taking at least one action to improve pollinator health in his/her landscape, yard or garden. Such actions include (a) plant selection to feed adults, (b) plants selection to feed larvae/young, (c) decrease/do not use pesticides, (d) add features to aid pollinator insects (brush piles, water sources, etc.), (e) Source plants locally, (f) primarily buy native plants, (g) primarily buy plants labeled as helpful to pollinators, (h) primarily buy flowering plants.

Figure 2a–c demonstrated that visual attention (i.e., fixation counts) were likely influenced by the participants’ knowledge and pollinator conservation activities, supporting Hypothesis 1 (similar patterns were found for total fixation duration (TFD); see Appendix A Figure A1a–c). Regarding neonicotinoid knowledge, not knowledgeable participants paid relatively more visual attention to the whole plant image, knowledgeable participants paid relatively more attention to neonicotinoid labels (Figure 2a). The differences were statistically significant, partially supporting Hypothesis 2a that a higher level of attribute-related knowledge will lead to greater visual attention to the associated attribute. Similarly, participants who were not knowledgeable about pollinator attractive plants, fixated more and for longer durations on the plant image compared to participants who were knowledgeable (Figure 2b). However, no significant difference was found for neonicotinoid labels between the two groups, indicating that Hypotheses 2a is only partially supported. Regarding engagement with pollinator conservation activities, participants who were not engaged in pollinator conservation activities paid more visual attention to all of the AOIs, but particularly more attention to the plant image, indicating no support for Hypothesis 2b (Figure 2c). 

Given the research’s focus on neonicotinoid labels, participants’ FCs to each of the neonicotinoid labels across plants was tabulated (Table 4; a summary of TFD and TFF is reported in Appendix A Table A3). Visual attention varied by the label’s format (logo vs. text) and framing. It ranged from a low of 34% (a11: neonic free logo) to as high as 72% (a2: protected by neonics). Among those who paid attention to neonicotinoid labels, participants spent more time reading the labels communicating the presence of neonicotinoids, indicated by more FCs and longer TFDs.

### 4.2. Econometric Model Estimates

The results of the random effects tobit model are summarized in Table 5 and Table 6 and Table A4 in the Appendix A. In general, participants valued labels disclosing the absence of neonicotinoids. Depending on the model specifications (i.e., incorporating relative total FCs, cascade effects, or relative TFD), participants were willing to pay a price premium of 20–29 cents for neonicotinoid-free text and 39–53 cents for the neonicotinoid-free logo relative to plants labeled with the “Protected from neonicotinoid” phrase (the base group). The coefficient of the neonicotinoid-treated variable was not statistically significant, indicating participants did not differentiate between the different texts communicating the presence of neonicotinoids. 

Participants were willing to pay about the same amount for a pentas plant, but 32–37 cents less for a marigold plant when comparted to impatiens. Participants were willing to pay higher amounts for the perennial plants, including 75–86 cents more for a dianthus plant, 1.12–1.73 dollars more for a chrysanthemum plant, and 59–72 cents more for a salvia plant than impatiens. Participants’ higher WTP for perennial plants may be attributed to the fact that perennial plants were in larger containers (i.e., one-gallon) and live longer.

Similar to Breeze et al. (2015) [46], individual sociodemographic variables (including age, gender, ethnicity, household size, education level, household income, allergies to pollen or to bee sting) were not statistically significant, and were suppressed from Table 5 and Table 6. Surprisingly, none of the three knowledge and conservation activity variables had a significant impact on participants’ bid value. This finding was consistent with Takahashi et al. (2018) [35], who also confirmed that participants’ awareness and previous experience did not influence their purchasing behaviors. Combined with the previous findings, this empirical evidence suggests that even though it is important to capture individual heterogeneity, individual characteristics were secondary to the product attributes in determining consumer preferences.

### 4.3. Evidence for Attribute Focus

To test for the effects of attribute focus on participants’ bidding behaviors, the relative total FCs (Table 5) and relative TFDs (Appendix A Table A4) were incorporated into the regression models. In general, evidence is limited that greater attention to specific attributes lead to positive or negative valuations depending on attribute characteristics. Specifically, increased focus on the plant image and container type did not alter participants’ bid value. Even though visual attention data revealed that participants paid greater attention to the plant images and container types, the results imply that greater attention to the plant image and container types did not factor into consumers’ bidding process. These results were in line with recent evidence [18,52,67]. 

Meanwhile, the impact of greater attention on neonicotinoid labels was asymmetric. While increased relative total FCs on neonicotinoid labels reduced bids for labels treated with neonicotinoid by 91 cents (supporting H3b), there was no effect on participants’ bids for neonicotinoid-free text and the neonicotinoid-free logo (not supporting H3a). The estimated results produced by relative TFDs was similar (Appendix A Table A4). Increased relative TFDs on neonicotinoid labels reduced participants’ bid values by 92 cents for neonicotinoid-treated labels but had no effect on the two labels disclosing the absence of neonicotinoids. In terms of the impact of attribute focus across knowledge or conservation involvement related groups, only participants engaged in pollinator conservation activities were likely to increase their bid value by about 94 cents (86 cents) if they fixated more (fixated longer) on the neonicotinoid labels, but not for the other groups. 

### 4.4. Gaze Cascade Effects

The results of the gaze cascade effect tests are reported in Table 6. Participants’ first and last fixations on the neonicotinoid labels did affect their bid values. The effect on bids was not observed for the plant image or container type. Specifically, if the first fixation was on a neonicotinoid label and the label disclosed the presence of neonicotinoids (e.g., treated with neonicotinoid), the participants were likely to bid 42 cents lower. Conversely, participants’ first fixation to labels disclosing the absence of neonicotinoids did not affect their bid value. In other words, the first fixation paid to neonicotinoid-free text and the neonicotinoid-free logo did not alter participants’ bid value, thus only partially supporting Hypothesis 4a. On the other hand, participants’ last fixation to a neonicotinoid label reduced participants’ bid values. For example, a last fixation to the neonicotinoid-free text label decreased bids by 46 cents, which was unexpected, and does not support Hypothesis 4b. Results suggest little evidence of initial or final tendencies to focus visual attention on a specific label/AOI, which could be correlated with participants’ decision making. In addition, we also tested the first and last cascade effects in relation to the positions of the AOIs. We grouped the four AOIs into two broader position categories: left and right. Left consisted of the plant name and neonicotinoid labels and the right consisted of the plant image and container type. The first and last gaze fixated on was tested to identify if the left or right side affected participants’ bid values. Similarly, very limited evidence was found that initial or final gaze on a specific area (left or right) affected participants’ decisions. The contents of the information and position of the information may jointly affect participant’s visual attention patterns, but not necessarily affect the decision making process (results are available from authors upon request). 

Even though different knowledge and pollinator conservation involvement based respondent groups showed distinct visual attention patterns, these differences in visual attention may or may not necessarily impact participants’ bidding decisions. Consistent with Orquin et al. (2018) [67], the regression results showed mixed messages about gaze cascade effects across the groups that differed in their knowledge and conservation practice involvement. For the self-perceived neonicotinoid knowledgeable group, there was no evidence that first/last fixation to the neonicotinoid labels or plant images led to changes in their bid values relative to their not knowledgeable counterparts. For participants knowledgeable about pollinator attractive plants, their first gaze on neonicotinoid labels decreased their bids by 31 cents, while their last gaze on the plant image increased their bids by 34 cents. For participants who were actively engaged in pollinator conservation activities, their last gaze fixated on neonicotinoid labels increased their bid value by 43 cents, mitigating the negative impact of the last gaze on neonicotinoid labels. 

## 5. Conclusions

Incorporating visual attention measures in consumer preference studies could provide useful information regarding participants’ decision making and bidding behavior. In general, participants valued labels disclosing the absence of neonicotinoids and were willing to pay a higher price premium for those products. Participants did not differentiate information formats when a plant was grown with neonicotinoids. This finding offers some interesting implications for the ongoing debate about mandatory disclosure of neonicotinoids on labels in the U.S. Given consumers’ positive response to products free of neonicotinoids, one anticipates a movement away from neonicotinoid use along the supply chain in the ornamental horticulture industry. In addition, policy makers may also consider a prudent policy approach by encouraging voluntary disclosure of the absence of neonicotinoids on labels since consumers prefer neonicotinoid free products. 

However, the link between visual attention and consumers’ attribute valuation and ultimate decisions is not necessarily strong. This study demonstrated that visual attention patterns were endogenous to individual knowledge about neonicotinoids and their engagement in pollinator conservation activities. The dichotomous groups exhibited distinct visual attention patterns in the process of determining their bids. While participants who self-reported as knowledgeable paid more attention to neonicotinoid labels, not knowledgeable participants paid more visual attention to the image of the (entire) product. Even though individual knowledge and activities about neonicotinoid were found to have no significant impact on participants’ WTP, leaving them in the error term would likely bias the estimated effect of visual attention on individual choice. This occurred because visual attention patterns were clearly correlated with individual knowledge and pollinator conservation activities, which reflect individual interests or concerns about pollinators. Further, policy makers may consider increasing public awareness of neonicotinoids and engaging consumers as part of a large pollinator conservation movement, which will likely change consumers’ attention on information, thus their shopping behaviors. 

There was some evidence of an asymmetric impact of attribute focus on consumers’ WTP. The negative impact of greater visual attention to negative features (i.e., treated with neonicotinoids) was evident in the present study. Specifically, even though consumers may have visually attended to both positive and negative information, it was the negative information that carried through to the final decision, leading to a stronger impact of negative information on consumer behavior identified by in information literature [60,61,62,63]. This finding provided supporting evidence for negativity effects of information. 

This finding may provide some insights on noncompensatory processing [66]. The results point to the possibility that the participants may have screened out product alternatives with undesired features. This process may play a larger role than strategies that consider alternatives with desired features in noncompensatory processing. First and last gaze cascade effect was confirmed by a significant negative impact of participants’ first fixation to labels disclosing the presence of neonicotinoids on their bid values. A first fixation on neonicotinoid labels decreased participants’ WTP for labels disclosing the presence of neonicotinoids (i.e., treated with neonicotinoid), but had no impact on their WTP for labels disclosing the absence of neonicotinoids (i.e., neonicotinoid-free text and logo). 

Further, the results provide additional support to Balcombe et al. (2017) [52] that the relationship between visual attention and preference could be weak. Participants may have visually attended to some (important) attributes; however, this information may or may not necessarily enter into their decision making process. On the other hand, it is possible that participants may have visually ignored certain (important) attributes which were actually used in their decision process. Van Loo et al. (2018) [18] recently acknowledged that it was more challenging to quantify attribute non-attendance using eye-tracking and to further infer the impact of visual attribute non-attendance on preference. 

There are several limitations to the present study. The endogeneity of visual attention data was approached using proxy variables to factor out the unobserved individual environmental interests from the error term. Further research could find valid instrument variables (IVs) for visual attention measures and use a two-stage estimation method such as 2SLS. Secondly, in contrast to previous findings (e.g., [35,88]) indicating that consumers place less value on the appearance of a food label logo, we found participants were willing to pay a higher price for a logo relative to the neonicotinoid-free text. Caution is needed when interpreting the logo price premiums. The “Better Bee Certified” logo does not explicitly display the information that is presented by the neonicotinoid-free attribute. Higher premiums for this logo could result from participants’ broader interpretation of this logo. Lastly, to test the first and last gaze cascade effect and circumvented the central fixation bias, the position of information (i.e., labels) was pre-set in the left-right paradigm. It is likely that the position and the content of the information jointly affect visual attention patterns, but we were not able to separate them in this study. Future studies may consider having control and treatment groups by switching the positions of labels to investigate which plays a larger role. Beyond these limitations, the results call for more attention to experimental design when using eye tracking and have implications for future studies to determine how to adequately use visual attention data in understanding its relationship with individual decision making. 

## Figures and Tables

**Table 1:** Plant attributes and attribute levels.

Attribute	Level 1	Level 2	Level 3	Level 4
Annual Bedding Plant Type	Impatiens (other)	Marigold	Pentas	---
Perennial Plant Type	Chrysanthemum	Dianthus	Salvia	---
Neonicotinoid Label	Neonicotinoid-Free (text)	Bee Better Certified (logo)	Treated with Neonicotinoids	Protected from Problematic Pests by Neonicotinoids
Container Type	Conventional Plastic	Bio-degradable	---	---

**Table 2:** Participants’ sociodemographic characteristics.

*Variable*	*Mean*
Number of participants	53
Male (%)	15%
Age (mean)	53.1
Household income (mean)	$40,000–$59,000
Plant purchase behavior	
Number of visits (mean)	6.4
Amount spend per visit (mean)	$37.0
Self-reported awareness of neonicotinoids (%)	22.6%
Self-perceived knowledge about neonicotinoids (%) ^a^	
Not knowledgeable	83.0%
Neither knowledgeable nor not knowledgeable	5.7%
Knowledgeable	11.3%
Self-perceived knowledge about pollinator attractive plants (%) ^a^	
Not knowledgeable	28.9%
Neither knowledgeable nor not knowledgeable	17.3%
Knowledgeable	53.9%
Real knowledge about pollinator attractive plants based on quiz questions (%) ^b^	
0 correct	7.7%
1 correct	23.1%
2 correct	30.8%
3 correct	32.7%
4 correct	5.8%
Pollinator conservation activities ^c^	
Doing nothing	18.9%
1~3 conservation activities	39.6%
More than 3 conservation activities	41.5%

^a^ Using a Likert scale ranging from 1 = Not at all knowledgeable to 7 = Extremely knowledgeable ratings, participants indicated how knowledgeable they were about neonicotinoid pesticides and pollinator attractive plants. Participants who selected 1 to 3, were combined into the category of “not knowledgeable” respondents. Participants who selected 4 were categorized as “neither knowledgeable nor not knowledgeable,” and participants who selected 5–7 were categorized as “knowledgeable.” ^b^ Participants answered four questions identifying pollinator attractive plants. ^c^ Participants were asked to select all the actions they were currently taking to improve pollinator health in their own landscape (e.g., yard, garden) from a pre-defined list (i.e., plant selection to feed adults, plant selection to feed larvae/young, decrease or do not use pesticides, add landscape features to aid pollinator insects, source plants locally, primarily buy native plants, primarily buy plants that are labeled as helpful to pollinators, and primarily buy flowering plants).

**Table 3:** Visual attention to plant name, neonicotinoid label, plant image, and container types.

		AOIs			
		Plant Name	Neonicotinoid Label	Plant Image	Container Types
**No. of observations**		1484	1484	1484	1484
**First Fixation (FF)**					
*(binary)*	Mean	0.141 (0.348)	0.182 (0.386)	0.363 (0.481)	0.094 (0.292)
**Last Fixation (LF)**					
*(binary)*	Mean.	0.155 (0.362)	0.100 (0.300)	0.123 (0.329)	0.420 (0.494)
**Fixation Counts (FC)**					
*(count)*	Mean	0.682 (1.254)	2.055 (3.474)	4.692 (7.062)	1.427 (2.229)
	Max	9	30	81	18
**Total Fixation Duration (TFD)**					
*(seconds)*	Mean	0.111(0.238)	0.346 (0.646)	0.937(1.627)	0.219 (0.417)
	Max	3.22	6.1	18.95	4.28
**Time to First Fixation (TFF)**					
*(seconds)*	Mean	2.809 (4.612)	2.805 (4.614)	2.467 (3.503)	3.408 (4.045)
	Max	49.74	60.97	30.91	42.2

Note: Standard deviation in parentheses.

**Table 4:** Summary of fixation counts to neonicotinoid labels.

Annual	Fixation Counts (FCs)		Perennial	Fixation Counts (FCs)		
	Mean	Std. Dev.		Mean	Std. Dev.	% ^a^
a1: Neonic free text	3.36	4.60	p1: Neonic free logo	1.94	3.02	60.4
a2: Protected by neonics	6.47	6.82	p2: Treated with neonics	1.81	2.89	71.7
a3: Neonic free text	2.91	4.09	p3: Neonic free text	1.43	1.92	66.0
a4: Protected by neonics	2.57	3.37	p4: Protected by neonics	4.02	5.55	60.4
a5: Neonic free text	1.91	2.79	P5: Protected by neonics	2.30	3.08	52.8
a6: Treated with neonics	1.87	2.69	p6: Neonic free text	2.28	4.03	62.3
a7: Treated with neonics	1.40	2.11	p7: Neonic free logo	1.45	2.74	49.1
a8: Neonic free logo	3.02	3.55	p8: Protected by neonics	2.45	3.76	64.2
a9: Neonic free logo	0.72	1.18	p9: Neonic free text	1.45	2.58	37.7
a10: Treated with neonics	1.09	1.78	p10: Neonic free logo	0.70	1.28	37.7
a11: Neonic free logo	0.62	1.10	p11: Treated with neonics	1.60	2.72	34.0
a12: Treated with neonics	1.57	2.37	p12: Neonic free text	0.87	1.66	45.3
a13: Neonic free text	1.74	2.93	p13: Treated with neonics	1.15	2.09	47.2
a14: Protected by neonics	3.25	4.83	p14: Treated with neonics	1.58	3.18	49.1

^a^ The percentage reports the number of participants having positive visual attention data (i.e., fixation count, total fixation duration) on the four types of neonicotinoid labels changing among alternatives.

**Table 5:** Effects of attribute focus (measured by relative total fixation counts) on bid value: random effects tobit model.

*Variables*	*Coefficients^e^*		*S.E.*	*Marginal Effects^f^*		*S.E.*
***Plant attributes***						
Neonicotinoid-free text(binary)^a^	0.331	^**^	0.143	0.202	^**^	0.093
Neonicotinoid-free logo (binary)^a^	0.596	^***^	0.148	0.387	^***^	0.100
Neonicotinoid-treated (binary)^a^	0.116		0.151	−0.100		0.099
Biodegradable pot	0.222	^***^	0.088	0.258	^***^	0.058
***Plant dummy^b^***						
Marigold	−0.362	^***^	0.122	−0.318	^***^	0.086
Pentas	−0.052		0.145	−0.117		0.095
Dianthus	0.975	^***^	0.150	0.758	^***^	0.095
Chrysanthemum	1.414	^***^	0.170	1.173	^***^	0.101
Salvia	0.851	^***^	0.206	0.635	^***^	0.106
***Individual heterogeneity^c^***						
Knowledge about neonicotinoids (binary)	−0.743		0.719	−0.522		0.582
Knowledge about p-attractive plants (binary)	0.218		0.504	0.188		0.409
Pollinator conservation (binary)	−0.371		0.610	−0.047		0.521
***Interaction Terms^d^***						
Neonicotinoid-free label * R_FC_neonic	−0.197		0.374	−0.177		0.336
Neonicotinoid-free logo * R_FC_neonic	−0.060		0.442	−0.054		0.397
Neonicotinoid-treated * R_FC_neonic	−1.015	^***^	0.372	−0.911	^***^	0.335
Biodegradable pot * R_FC_pot	0.257		0.268	0.231		0.240
Plant * R_FC_plant	−0.081		0.058	−0.073		0.052
Knowledge about neonics *R_FC_neonic	0.067		0.539	0.060		0.484
Knowledge about p-attractive plants * R_FC_neonic	−0.532		0.388	−0.478		0.348
Pollinator conservation *R_FC_neonic	1.047	^***^	0.341	0.940	^***^	0.307
Knowledge about neonics *R_FC_plant	0.153		0.501	0.137		0.450
Knowledge about p-attractive plants * R_FC_plant	0.365		0.338	0.327		0.303
Pollinator conservation *R_FC_plant	0.298		0.314	0.268		0.282
Constant	1.049		1.439	-		-
$\sigma_{c}$	1.338	^***^	0.151	-		-
$\sigma_{u}$	1.070	^***^	0.026	-		-
ρ	0.610		0.055	-		-
No. of observations	1021					
Log likelihood	−1552.16					

^a^ Labels with text “*Protected from Problematic Pests by Neonicotinoids*” was used as the base group. Variable Neonicotinoid-free_text equals 1 if labeled with text “*Neonicotinoid-Free,*” 0 otherwise. Variable Neonicotinoid-free_logo equals 1 if labeled with logo “*Bee Better Certified,*” 0 otherwise. Variable Neonicotinoid-treated equals 1 if labeled with text “*Treated with Neonicotinoids.*” ^b^ Annual bedding plant Impatiens was used as the base group. ^c^ Other individual characteristics (including age, gender, ethnicity, household size, education level, household income, whether or allergic to pollen and whether allergic to sting) were controlled. Estimated results were suppressed in the table. ^d^ R_FC stood for relative fixation counts on neonicotinoid labels (R_FC_neonic), container type (R_FC_pot) and plant image R_FC_plant). ^e^ ** and *** indicate the coefficients are statistically significant (** p < 0.05; and *** p < 0.01) ^f^ Marginal effects on the observed variables were reported in this table, which were the unconditional partial effects averaged across all sample observations.

**Table 6:** Gaze cascade effect on choice: random effects tobit model.

Variables	*Coefficients^e^*		*S.E.*	*Marginal Effects^f^*		*S.E.*
***Plant attributes***						
Neonicotinoid-free text(binary)^a^	0.248	^**^	0.102	0.222	^**^	0.091
Neonicotinoid-free logo (binary)^a^	0.475	^***^	0.106	0.426		0.096
Neonicotinoid-treated (binary)^a^	−0.048		0.107	−0.043		0.096
Biodegradable pot	0.264	^***^	0.068	0.236	^***^	0.061
***Plant dummy^b^***						
Marigold	−0.436	^***^	0.104	−0.367	^***^	0.089
Pentas	−0.156		0.116	−0.134		0.100
Dianthus	0.819	^***^	0.113	0.745	^***^	0.104
Chrysanthemum	1.215	^***^	0.124	1.123	^***^	0.116
Salvia	0.648	^***^	0.139	0.585	^***^	0.126
***Individual heterogeneity^c^***						
Knowledge about neonicotinoids (binary)	−0.921		0.643	−0.824		0.573
Knowledge about p-attractive plants (binary)	0.389		0.452	0.349		0.404
Pollinator conservation (binary)	−0.047		0.573	−0.042		0.513
***Interaction Terms^d^***						
Neonicotinoid-free label * FF_neonic	0.237		0.194	0.212		0.174
Neonicotinoid-free logo * FF_neonic	0.268		0.237	0.240		0.212
Neonicotinoid-treated * FF_neonic	−0.465	^***^	0.195	−0.416	^**^	0.175
Biodegradable pot * FF_Pot	−0.032		0.143	−0.029		0.128
Plant * FF_plant	−0.013		0.030	−0.012		0.027
Neonicotinoid-free label * LF_neonic	−0.517	^**^	0.248	−0.462	^**^	0.222
Neonicotinoid-free logo * LF_neonic	−0.413		0.264	−0.370		0.237
Neonicotinoid-treated * LF_neonic	−0.128		0.224	−0.115		0.200
Biodegradable pot * LF_pot	−0.021		0.123	−0.019		0.116
Plant *LF_plant	0.017		0.029	0.015		0.026
Knowledge about neonics *FF_neonic	0.347		0.239	0.310		0.214
Knowledge about p-attractive plants * FF_neonic	−0.349	^**^	0.175	−0.313	^**^	0.157
Pollinator conservation *FF_neonic	0.105		0.168	0.094		0.150
Knowledge about neonics *LF_neonic	−0.183		0.279	−0.164		0.249
Knowledge about p-attractive plants * LF_neonic	0.118		0.200	0.106		0.179
Pollinator conservation *LF_neonic	0.429	^**^	0.193	0.384	^**^	0.173
Knowledge about neonics *FF_plant	0.136		0.235	0.122		0.210
Knowledge about p-attractive plants * FF_plant	−0.443	^***^	0.156	−0.397	^***^	0.140
Pollinator conservation *FF_plant	0.179		0.142	0.160		0.127
Knowledge about neonics *LF_plant	0.315		0.217	0.282		0.195
Knowledge about p-attractive plants * LF_plant	0.339	^**^	0.143	0.304	^**^	0.129
Pollinator conservation *LV_plant	−0.169		0.136	−0.151		0.122
Constant	0.974		1.412	-		-
$\sigma_{c}$	1.328	^***^	0.146	-		-
$\sigma_{u}$	1.033	^***^	0.022	-		-
ρ	0.623		0.053	-		-
No. of observations	1266					
Log likelihood	−1849.23					

^a^ Labels with text “Protected from Problematic Pests by Neonicotinoids” was used as the base group. Variable Neonicotinoid-free_text equals 1 if labeled with text “Neonicotinoid-Free,” 0 otherwise. Variable Neonicotinoid-free_logo equals 1 if labeled with logo “Bee Better Certified,” 0 otherwise. Variable Neonicotinoid-treated equals 1 if labeled with text “Treated with Neonicotinoids.” ^b^ Annual bedding plant Impatiens was used as the base group. ^c^ Other individual characteristics (including age, gender, ethnicity, household size, education level, household income, whether or allergic to pollen and whether allergic to sting) were controlled. Estimated results were suppressed in the table. ^d^ FF and LF stood for first fixation and last fixation on neonicotinoid labels (FF_neonic, LF_neonic), container type (FF_pot, LF_pot) and plant image (FF_plant, LF_plant). ^e^ ** and *** indicate the coefficients are statistically significant (** p < 0.05; and *** p < 0.01). ^f^ Marginal effects on the observed variables were reported in this table, which were the unconditional partial effects averaged across all sample observations.

## Appendix A. Tables and Figures

**Table A1:** Correlations among individual knowledge and involvement variables.

	Perceived Knowledge about Neonicotinoids	Perceived Knowledge about Pollinator Attractive Plants	Real Knowledge about Pollinator Attractive Plants	Pollinator Conservation Activities
Perceived knowledge about neonicotinoids	1.000			
Perceived knowledge about pollinator attractive plants	0.433	1.000		
Real knowledge about pollinator attractive plants	0.141	0.121	1.000	
Pollinator conservation activities	0.269	0.437	0.252	1.000

**Table A2:** Age and gender comparison across knowledge and involvement variables.

	No. of Obs.	Age (Mean)	No. of Obs.	Gender (Mean)
Perceived knowledge about neonicotinoids				
Not knowledgeable (0)	42	50.24	44	0.16
Knowledgeable (1)	8	66.63	8	0.13
Difference(0–1)	−16.39 *t* = −2.89 (*p*-value = 0.01)		0.03 *t* = −0.24 (*p*-value = 0.81)	
Revealed knowledge about pollinator attractive plants				
Not knowledgeable (0)	29	51.72	31	0.16
Knowledgeable (1)	20	56	20	0.1
Difference(0–1)	−4.28 *t* = −0.96 (*p*-value = 0.34)		0.06 *t* = −0.61 (*p*-value = 0.54)	
Pollinator conservation activity				
Not involved (0)	10	50.3	10	0.2
Involved (1)	40	53.5	42	0.14
Difference(0–1)	−3.2 *t* = −0.057 (*p*-value = 0.57)		0.06 *t* = −0.44 (*p*-value = 0.66)	

**Table A3:** Summary of TFD and TFF patterns to neonicotinoid labels.

**Annual**	**Total Fixation Duration (TFD)**		**Time to First Fixation (TFF)**		
	**Mean**	**Std. Dev.**	**Mean**	**Std. Dev.**	**%^a^**
a1: Neonic free text	1.07	1.19	5.30	7.61	60.4
a2: Protected by neonics	1.59	1.28	1.85	1.55	71.7
a3: Neonic free text	0.76	0.81	3.07	5.15	66.0
a4: Protected by neonics	0.68	0.67	2.26	2.62	60.4
a5: Neonic free text	0.59	0.54	3.50	4.20	52.8
a6: Treated with neonics	0.45	0.48	3.08	3.22	62.3
a7: Treated with neonics	0.43	0.35	2.06	2.03	49.1
a8: Neonic free logo	0.83	0.75	3.39	8.37	64.2
a9: Neonic free logo	0.29	0.24	1.85	1.81	37.7
a10: Treated with neonics	0.45	0.33	2.43	2.08	37.7
a11: Neonic free logo	0.28	0.23	3.15	3.97	34.0
a12: Treated with neonics	0.53	0.36	1.66	1.50	45.3
a13: Neonic free text	0.66	0.88	2.30	2.63	47.2
a14: Protected by neonics	1.05	0.83	1.76	2.16	49.1
**Perennial**	**Total Fixation Duration (TFD)**		**Time to First Fixation (TFF)**		
	**Mean**	**Std. Dev.**	**Mean**	**Std. Dev.**	**%^a^**
p1: Neonic free logo	0.70	0.70	7.83	13.14	49.1
p2: Treated with neonics	0.58	0.54	3.70	3.32	50.9
p3: Neonic free text	0.44	0.39	1.81	1.23	54.7
p4: Protected by neonics	1.13	1.07	2.05	3.40	58.5
P5: Protected by neonics	0.67	0.54	2.24	2.70	54.7
p6: Neonic free text	0.75	1.00	1.55	1.37	58.5
p7: Neonic free logo	0.66	0.77	3.76	3.48	43.4
p8: Protected by neonics	0.79	0.67	2.67	3.15	49.1
p9: Neonic free text	0.60	0.61	1.83	2.95	39.6
p10: Neonic free logo	0.33	0.23	2.99	2.97	30.2
p11: Treated with neonics	0.55	0.52	2.56	4.21	50.9
p12: Neonic free text	0.45	0.44	2.88	4.90	34.0
p13: Treated with neonics	0.41	0.38	2.00	2.40	39.6
p14: Treated with neonics	0.47	0.67	2.72	4.60	54.7

^a^ The percentage reports the number of participants having positive visual attention data (i.e., fixation count, total fixation duration) on the four types of neonicotinoid labels changing among alternatives.

**Table A4:** Effects of attribute focus (measured by relative total fixation duration) on bid value: random effects tobit model.

Variables	*Coefficients^e^*		*S.E.*	*Marginal Effects^f^*		*S.E.*
***Plant attributes***						
Neonicotinoid-free text(binary)^a^	0.320	^**^	0.140	0.288	^**^	0.126
Neonicotinoid-free logo (binary)^a^	0.595	^***^	0.144	0.534	^***^	0.130
Neonicotinoid-treated (binary)^a^	0.111		0.148	0.099		0.133
Biodegradable pot	0.217	^**^	0.085	0.195	^**^	0.077
***Plant dummy^b^***						
Marigold	−0.375	^***^	0.123	−0.313	^***^	0.104
Pentas	−0.078		0.145	−0.067		0.124
Dianthus	0.944	^***^	0.152	0.858	^***^	0.138
Chrysanthemum	1.375	^***^	0.173	1.270	^***^	0.159
Salvia	0.798	^***^	0.210	0.721	^***^	0.190
***Individual heterogeneity^c^***						
Knowledge about neonicotinoids (binary)	−0.784		0.715	−0.704		0.641
Knowledge about p-attractive plants (binary)	0.157		0.504	0.141		0.453
Pollinator conservation (binary)	−0.349		0.610	−0.314		0.548
***Interaction Terms^d^***						
Neonicotinoid-free label * R_TFD_neonic	−0.181		0.365	−0.163		0.328
Neonicotinoid-free logo * R_TFD _neonic	−0.114		0.429	−0.103		0.386
Neonicotinoid-treated * R_TFD_neonic	−1.029	^***^	0.368	−0.924	^***^	0.331
Biodegradable pot * R_TFD _pot	0.319		0.272	0.287		0.244
Plant * R_TFD _plant	−0.061		0.057	−0.055		0.051
Knowledge about neonics * R_TFD_neonic	0.125		0.535	0.112		0.480
Knowledge about p-attractive plants * R_TFD _neonic	−0.348		0.387	−0.312		0.348
Pollinator conservation *R_TFD _neonic	0.955	^***^	0.339	0.858	^***^	0.305
Knowledge about neonics * R_TFD_plant	0.224		0.482	0.201		0.433
Knowledge about p-attractive plants * R_TFD_plant	0.388		0.335	0.348		0.301
Pollinator conservation * R_TFD_plant	0.291		0.309	0.261		0.277
Constant	1.040		1.431	-		-
$\sigma_{c}$	1.330		0.150	-		-
$\sigma_{u}$	1.072		0.026	-		-
ρ	0.606		0.055	-		-
No. of observations	1201					
Log likelihood	−1553.16					

^a^ Labels with text “*Protected from Problematic Pests by Neonicotinoids*” was used as the base group. Variable Neonicotinoid-free_text equals 1 if labeled with text “*Neonicotinoid-Free*,” 0 otherwise. Variable Neonicotinoid-free_logo equals 1 if labeled with logo “*Bee Better Certified,*” 0 otherwise. Variable Neonicotinoid-treated equals 1 if labeled with text “*Treated with Neonicotinoids.*” ^b^ Annual bedding plant Impatiens was used as the base group. ^c^ Other individual characteristics (including age, gender, ethnicity, household size, education level, household income, whether or allergic to pollen and whether allergic to sting) were controlled. Estimated results were suppressed in the table. ^d^ R_TFD stood for relative total fixation duration on neonicotinoid labels (R_TFD_neonic), container type (R_TFD_pot) and plant image R_TFD_plant). ^e^ ** and *** indicate the coefficients are statistically significant (** p < 0.05; and *** p < 0.01). ^f^ Marginal effects on the observed variables were reported in this table, which were the unconditional partial effects averaged across all sample observations.
